# Arginine supplementation modulates pig plasma lipids, but not hepatic fatty acids, depending on dietary protein level with or without leucine

**DOI:** 10.1186/s12917-017-1063-y

**Published:** 2017-05-30

**Authors:** Marta Sofia Morgado dos Santos Madeira, Eva Sofia Alves Rolo, Virgínia Maria Rico Pires, Cristina Maria Riscado Pereira Mateus Alfaia, Diogo Francisco Maurício Coelho, Paula Alexandra Antunes Brás Lopes, Susana Isabel Vargas Martins, Rui Manuel Amaro Pinto, José António Mestre Prates

**Affiliations:** 1 CIISA, Faculdade de Medicina Veterinária, Universidade de Lisboa, Avenida da Universidade Técnica, Pólo Universitário do Alto da Ajuda, 1300-477 Lisbon, Portugal; 2 iMed.UL, Faculdade de Farmácia, Universidade de Lisboa, Avenida Professor Gama Pinto, 1649-003 Lisbon, Portugal

**Keywords:** Arginine, Leucine, Low dietary protein, Fatty acids, Gene expression, Liver, Pig

## Abstract

**Background:**

In the present study, the effect of arginine and leucine supplementation, and dietary protein level, were investigated in commercial crossbred pigs to clarify their individual or combined impact on plasma metabolites, hepatic fatty acid composition and mRNA levels of lipid sensitive factors. The experiment was conducted on fifty-four entire male pigs (Duroc × Pietrain × Large White × Landrace crossbred) from 59 to 92 kg of live weight. Each pig was randomly assigned to one of six experimental treatments (*n* = 9). The treatments followed a 2 × 3 factorial arrangement, providing two levels of arginine supplementation (0 vs. 1%) and three levels of basal diet (normal protein diet, NPD; reduced protein diet, RPD; reduced protein diet with 2% of leucine, RPDL).

**Results:**

Significant interactions between arginine supplementation and protein level were observed across plasma lipids. While dietary arginine increased total lipids, total cholesterol, HDL-cholesterol, LDL-cholesterol, VLDL-cholesterol and triacylglycerols in NPD, the inverse effect was observed in RPD. Overall, dietary treatments had a minor impact on hepatic fatty acid composition. RPD increased 18:1*c*9 fatty acid while the combination of leucine and RPD reduced 18:0 fatty acid. Arginine supplementation increased the gene expression of *FABP1*, which contributes for triacylglycerols synthesis without affecting hepatic fatty acids content. RPD, with or without leucine addition, upregulated the lipogenic gene *CEBPA* but downregulated the fat oxidation gene *LPIN1*.

**Conclusions:**

Arginine supplementation was responsible for a modulated effect on plasma lipids, which is dependent on dietary protein level. It consistently increased lipaemia in NPD, while reducing the correspondent metabolites in RPD. In contrast, arginine had no major impact, neither on hepatic fatty acids content nor on fatty acid composition. Likewise, leucine supplementation of RPD, regardless the presence of arginine, promoted no changes on total fatty acids in the liver. Ultimately, arginine, leucine and dietary protein reduction seem to be unrelated with fatty liver development.

## Background

Pork is the most consumed meat in European Union countries [1]. Accordingly, swine research has been focused on the improvement of meat quality and growth performance parameters in the last decades. The genetic selection of commercial pig lines has reduced subcutaneous fat content while decreasing simultaneously the intramuscular fat (IMF) content. IMF is a key meat quality trait, and the sensory properties of pork are negatively affected when IMF drops below 2–2.5% [2]. Moreover, due to anatomic and physiologic similarities with humans, pig has been used as an excellent biomedical model to study a wide range of human health conditions [3], in particular concerning nutrient digestion, absorption and metabolism [4, 5].

Some feeding strategies based on dietary amino acid supplementation and reduced protein diets (RPD) have been suggested to improve fat partitioning in pigs [6–8], that is, to increase IMF content which contributes to improve pork sensory traits, such as tenderness and juiciness, without affecting backfat thickness. In addition, arginine is classified as a nonessential amino acid for healthy adults because it is synthesised in most mammals, including humans, pigs or rats [9, 10]. Arginine plays multiple physiological functions in animals, one of which is the ability to enhance lipolysis through the expression of key genes responsible for fatty acid oxidation in a tissue-specific manner [11, 12]. Recently, it has been reported that arginine supplementation reduces fat accumulation in white adipose tissue of obese models, such as humans [13], rats [14], sheep [15] and growing finishing pigs [16].

Moreover, the essential amino acid leucine plays a pivotal role in protein synthesis in the muscle [17]. Some studies suggested that diets with high levels of leucine can increase IMF content in finishing pigs [6]. In addition to the supplementation of dietary amino acids, arginine and leucine, the use of RPD for increasing IMF content in pigs, with less effect on subcutaneous fat deposition, has also been described [7]. Donato et al. [18] reported that leucine supplementation during caloric restriction in rats also results in more fat loss and improves protein synthesis in both liver and muscle. Nonetheless, the effect of arginine and leucine supplemented to low protein diets on hepatic fatty acid metabolism remains elusive.

An experiment with fifty-four commercial crossbred male pigs fed on normal and reduced protein diets, with or without arginine and leucine addition, was carried out to study the effect, individual or combined, of dietary protein level and amino acids supplementation on lipid metabolism of adipose tissue and skeletal muscle. We reported previously that dietary arginine supplementation does not have a significant effect on IMF content, but increased total fat in subcutaneous adipose tissue [19]. However, some studies with dietary supplementation [8, 20] found an increase in IMF content without changing pork quality. Thus, arginine might be involved in the differential regulation of some key lipogenic genes expression in pig’s muscle and subcutaneous adipose tissue [21]. RPD increased IMF content and total fat content in subcutaneous adipose tissue. Moreover, leucine supplementation on RPD does not seem to result in an additional increase of IMF and total fat in subcutaneous adipose tissue [22]. These results suggest that adipogenesis and lipogenesis might be differently regulated in pig’s *longissimus lumborum* muscle and subcutaneous adipose tissue [19]. In the present study, and following on the same animal trial [19], we hypothesised that dietary arginine supplementation, RPD and RPD with leucine promote hepatic lipogenesis in pigs. To test this hypothesis, we assessed the fatty acid content and composition, as well as the gene expression levels of essential lipogenic enzymes and associated transcription factors.

## Results

The present study reports the additive effect of dietary arginine and reduced protein diets, with or without leucine supplementation, on hepatic fatty acid composition and transcriptional profile of key lipogenic enzymes and transcription factors, using commercial crossbred pigs. This experiment also produced results on pigs’ performance and carcass traits that are presented elsewhere [19]. In brief, low-protein diets decrease animal performance in lean pigs, while dietary arginine has no effect on growth parameters [22]. Moreover, dietary leucine on low-protein diets does not seem to play any additional effect on pig growth performance or pork quality traits.

### Plasma metabolites

The biochemical profile in plasma is shown in Table 1. A significant interaction between arginine supplementation and protein level (*P* < 0.001) was consistently observed across plasma lipids. Total lipids increased with RPD but decreased with RPDL, and also increased with arginine supplementation but decreased with arginine combined with RPD or RPDL (*P* < 0.001). The same variations were found for triacylglycerols (*P* < 0.001). Total cholesterol, HDL-cholesterol and LDL-cholesterol increased with RPD and with arginine supplementation (*P* < 0.001). Also, the RPD in combination with arginine decreased total cholesterol (*P* < 0.001). In addition, HDL-cholesterol decreased with arginine in combination with RPD, but increased with leucine (*P* < 0.001). This change contrasts with LDL-cholesterol, which decreased with arginine combined with RPD and leucine (*P* < 0.001). VLDL-cholesterol increased with RPD, but decreased with RPDL (*P* < 0.001). Also, arginine supplementation increased VLDL-cholesterol, but in combination with RPD decreased its levels (*P* < 0.001), which in turn were increased with leucine (*P* < 0.001).

**Table 1:** Effect of dietary arginine, leucine and protein level on plasma metabolites of commercial crossbred pigs^1^ ^−3^

	Control						Arginine						Significance level				
	NPD		RPD		RPDL		NPD		RPD		RPDL		Arginine	Dietary protein level			Arg × Prot
	Mean	SE	Mean	SE	Mean	SE	Mean	SE	Mean	SE	Mean	SE		NPD vs. RPD	NPD vs. RPDL	RPD vs. RPDL	
Plasma lipids																	
Total lipids (mg/L)	3691^c^	19	4177^a^	27	4040^b^	29	4114^ab^	23	3678^c^	27	3735^c^	24	<0.001	0.320	0.536	0.151	<0.001
Triacylglycerols (mg/L)	398^b^	5.5	441^a^	11	291^cd^	18	421^a^	6.1	304^d^	8.3	329^c^	6.5	0.007	<0.001	<0.001	<0.001	<0.001
Total Cholesterol (mg/L)	897^c^	8.7	1118^ab^	13.9	1124^a^	8.8	1097^a^	10.1	937^b^	12.9	953^b^	9.7	<0.001	0.013	<0.001	0.321	<0.001
HDL-cholesterol (mg/L)	328^d^	9.4	405^ab^	5.5	417a	6.0	385^c^	6.0	335^d^	3.8	391^bc^	4.8	0.017	0.044	<0.001	<0.001	<0.001
LDL-cholesterol (mg/L)	490^c^	10	601^ab^	27	649^a^	10	628^a^	11	541^b^	14	495^c^	6.5	0.049	0.496	0.194	0.946	<0.001
VLDL-cholesterol (mg/L)	79.5^b^	1.09	88.2^a^	3.72	58.2^cd^	2.24	84.2^a^	1.22	60.9^d^	1.67	65.8^c^	1.31	0.007	<0.001	<0.001	<0.001	<0.001
Other plasma metabolites																	
Glucose (mg/L)	1540^a^	28	1150^c^	20	1210^bc^	25	1160^c^	19	1170^c^	18	1230^b^	16	<0.001	<0.001	<0.001	0.007	<0.001
Insulin (mU/L)	3.71	0.458	3.48	0.458	3.77	0.458	3.05	0.458	3.36	0.458	2.68	0.458	0.102	0.933	0.736	0.674	0.581
HOMA-IR^3^ (mmol/l × μU/ml)	1.40	0.206	0.99	0.171	1.13	0.161	0.88	0.122	0.96	0.103	0.81	0.084	0.021	0.304	0.269	0.963	0.283
Leptin (μg/L)	1.25	0.134	1.49	0.134	1.92	0.134	1.40	0.134	1.61	0.134	1.54	0.134	0.718	0.096	0.004	0.190	0.088
Urea (mg/L)	234^b^	7.7	274^a^	9.4	217^bc^	7.8	222^b^	5.9	177^d^	5.3	200^c^	5.5	<0.001	0.705	0.006	0.025	<0.001
Total protein (g/L)	71.3^b^	0.40	74.3^a^	0.57	67.7^c^	0.77	71.4^b^	0.32	66.7^c^	0.79	66.7^c^	0.19	0.612	0.134	0.297	0.040	<0.001
Plasma hepatic markers																	
ALT (U/L)	47.3^b^	0.60	48.9^b^	0.54	42.9^c^	0.54	53.0^a^	1.49	40.7^c^	1.94	33.9^d^	1.80	0.001	0.001	<0.001	<0.001	<0.001
AST (U/L)	57.4	1.25	48.8	0.64	36.1	1.73	53.0	1.19	42.4	1.79	36.5	1.92	0.007	<0.001	<0.001	<0.001	0.122
GGT (U/L)	18.8^d^	0.43	26.7^bc^	1.82	29.5^b^	0.58	28.3^bc^	1.07	25.5^c^	1.38	42.4^a^	1.05	<0.001	0.059	<0.001	<0.001	<0.001

^1^ *NPD* normal protein diet, *RPD* reduced protein diet, *RPDL* reduced protein diet with leucine addition.

^2^ *AST* aspartate aminotransferase (EC 2.6.1.1), *ALT* alanine aminotransferase (EC 2.6.1.2), *ALP* alkaline phosphatase (EC 3.1.3.1), *GGT* gamma-glutamyltransferase (EC 2.3.2.13).

^3^HOMA-IR, insulin resistance index = [fasting plasma glucose] × [fasting plasma insulin] / 22.5.

(a-d) mean values within a row with unlike superscript letter were significantly different (P < 0.05)

A significant interaction between arginine and protein level was found for glucose (*P* < 0.001), urea (*P* < 0.001) and total protein (*P* < 0.001). Glucose decreased with RPD and arginine supplementation, but arginine with RPDL increased its value. Urea increased with RPD, but when combined with leucine decreased its value when compared to the RPD. Arginine supplementation increased urea levels in RPDL; the inverse effect was observed in RPD. Curiously, arginine has no effect in NPD. Total protein increased with RPD and decreased with RPDL. Arginine had no impact on total protein, but arginine on RPD decreased its values. Arginine decreased HOMA-IR (*P* = 0.021). For plasma hormones, only leptin increased with RPDL when compared to NPD (*P* = 0.004).

Regarding plasma hepatic markers, a significant interaction between arginine supplementation and protein level was found for ALT (*P* < 0.001) and GGT (*P* < 0.001). Arginine supplementation increased ALT and GGT in NPD. RPDL, as well as arginine, on RPD and RPDL decreased ALT. RPD, RPDL and RPDL with arginine supplementation increased GGT. RPD (*P* < 0.001), RPDL (*P* < 0.001) and arginine supplementation decreased AST (*P* = 0.007).

### Total lipids and fatty acid composition in the liver

Lipid content and fatty acid composition determined in the liver are shown in Table 2. Dietary treatments had no impact on total fatty acid content (*P* > 0.05). The prevalent fatty acids found across dietary groups were 18:0 (26–29%), 16:0 (16–18%), 18:2*n*-6 (14–17%), 18:1*c*9 (13–16%) and 20:4*n-*6 (9–11% of total FAME). Arginine supplementation affected only 2 of the 24 fatty acids identified. The proportions of 15:0 (*P* = 0.002) and 20:0 (*P* = 0.035) were increased in pigs fed on dietary arginine. The 15:0 proportion decreased with RPDL when compared to NPD (*P* = 0.013) and RPD (*P* = 0.019). 18:0 decreased (*P* = 0.035) with RPDL when compared to NPD. The 18:1*c*9 proportion increased (*P* = 0.028) with RPD when compared to the NPD. Neither fatty acid partial sums nor ratios were affected by dietary treatments (*P* > 0.05).

**Table 2:** Effect of dietary arginine, leucine and protein level on total fatty acids and fatty acid composition in the liver of commercial crossbred pigs^1−3^

	Control							Arginine					Significance level				
	NPD		RPD		RPDL		NPD		RPD		RPDL		Arginine	Dietary protein level			Arg × Prot
	Mean	SE	Mean	SE	Mean	SE	Mean	SE	Mean	SE	Mean	SE		NPD vs. RPD	NPD vs. RPDL	RPD vs. RPDL	
*TFA*	1.47	0.094	1.45	0.094	1.31	0.094	1.31	0.094	1.25	0.094	1.25	0.094	0.075	0.246	0.641	0.484	0.725
*Fatty acid composition*																	
12:0	0.13	0.007	0.17	0.036	0.15	0.009	0.16	0.012	0.15	0.012	0.15	0.007	0.577	0.539	0.607	0.801	0.151
14:0	0.29	0.034	0.35	0.034	0.38	0.034	0.31	0.034	0.35	0.034	0.33	0.034	0.677	0.096	0.098	0.992	0.546
15:0	0.15	0.045	0.31	0.045	0.24	0.045	0.36	0.045	0.43	0.045	0.28	0.045	0.002	0.889	0.013	0.019	0.170
16:0	16.2	0.631	17.0	0.631	18.3	0.631	17.1	0.631	17.0	0.631	16.9	0.631	0.696	0.141	0.588	0.347	0.214
16:1*c*7	0.36	0.031	0.40	0.031	0.42	0.031	0.38	0.031	0.37	0.031	0.39	0.031	0.674	0.210	0.606	0.456	0.712
16:1*c*9	0.55	0.064	0.61	0.064	0.67	0.064	0.50	0.064	0.62	0.064	0.58	0.064	0.408	0.132	0.159	0.918	0.743
17:0	1.07	0.126	1.36	0.126	1.28	0.126	1.28	0.126	0.35	0.126	1.25	0.126	0.553	0.471	0.154	0.473	0.592
17:1*c*9	0.20	0.021	0.19	0.021	0.19	0.021	0.16	0.021	0.18	0.021	0.18	0.021	0.227	0.790	0.709	0.915	0.860
18:0	28.4	0.97	26.1	0.97	26.3	0.97	29.5	0.97	27.5	0.97	27.9	0.97	0.093	0.068	0.035	0.766	0.972
18:1*c*9	13.6	0.86	15.0	0.86	16.1	0.86	13.7	0.86	14.3	0.86	15.0	0.86	0.443	0.028	0.241	0.289	0.810
18:1*c*11	1.80	0.086	1.65	0.086	1.69	0.086	1.75	0.086	1.64	0.086	1.71	0.086	0.896	0.387	0.149	0.555	0.910
18:2*n*-6	16.8	0.88	16.1	0.88	14.3	0.88	14.5	0.88	16.1	0.88	14.9	0.88	0.427	0.229	0.632	0.095	0.222
18:3*n*-3	0.32	0.031	0.27	0.031	0.27	0.031	0.26	0.031	0.32	0.031	0.25	0.031	0.739	0.289	0.901	0.245	0.257
20:0	0.067	0.005	0.061	0.005	0.062	0.005	0.079	0.005	0.068	0.005	0.070	0.005	0.035	0.191	0.104	0.741	0.843
20:1*c*11	0.21	0.015	0.22	0.015	0.25	0.015	0.23	0.015	0.21	0.015	0.24	0.015	0.689	0.095	0.763	0.050	0.417
20:2*n*-6	0.48	0.031	0.57	0.074	0.45	0.074	0.45	0.074	0.50	0.032	0.46	0.031	0.363	0.744	0.154	0.093	0.577
20:3*n*-3	0.59	0.063	0.60	0.063	0.51	0.063	0.44	0.063	0.51	0.063	0.49	0.063	0.086	0.813	0.523	0.382	0.579
20:3*n*-6	0.47	0.089	0.48	0.089	0.49	0.064	0.42	0.068	0.53	0.023	0.48	0.061	0.977	0.570	0.350	0.727	0.664
20:4*n*-6	10.5	1.27	9.82	1.27	9.75	1.27	8.59	1.27	10.5	1.27	10.2	1.27	0.794	0.743	0.634	0.882	0.532
20:5*n*-3	0.40	0.093	0.41	0.093	0.51	0.093	0.65	0.093	0.51	0.093	0.55	0.093	0.092	0.948	0.510	0.469	0.493
22:0	0.52	0.103	0.48	0.103	0.56	0.103	0.74	0.103	0.57	0.103	0.62	0.103	0.158	0.712	0.309	0.514	0.682
22:4*n*-6	0.67	0.139	0.67	0.139	0.83	0.139	0.64	0.139	0.65	0.139	0.70	0.139	0.580	0.426	0.981	0.440	0.899
22:5*n*-3	1.07	0.185	1.03	0.185	1.00	0.185	0.79	0.185	1.05	0.185	0.96	0.185	0.526	0.785	0.558	0.753	0.696
22:6*n*-3	0.74	0.120	0.59	0.120	0.46	0.120	0.60	0.120	0.54	0.120	0.56	0.120	0.783	0.180	0.384	0.631	0.611
Others	3.66	0.197	4.76	0.882	3.98	0.258	5.69	1.215	3.15	0.252	3.95	0.246	0.808	0.294	0.361	0.981	0.097
*Fatty acid partial sums*																	
SFA	47.1	1.41	46.1	1.41	47.6	1.41	49.8	1.41	47.8	1.41	47.8	1.41	0.205	0.612	0.302	0.596	0.675
MUFA	16.7	1.02	18.1	1.02	19.3	1.02	16.7	1.02	17.4	1.02	18.2	1.02	0.449	0.051	0.326	0.316	0.857
PUFA	32.1	2.24	30.6	2.24	28.6	2.24	27.4	2.24	31.2	2.24	29.6	2.24	0.577	0.769	0.609	0.422	0.368
*n*-6 PUFA	29.0	2.00	27.7	2.00	25.8	2.00	24.6	2.00	28.3	2.00	26.8	2.00	0.568	0.800	0.564	0.407	0.344
*n*-3 PUFA	3.12	0.257	2.90	0.257	2.75	0.257	2.75	0.257	2.94	0.257	2.81	0.257	0.678	0.557	0.966	0.586	0.644
*Fatty acid ratios*																	
PUFA/SFA	0.69	0.068	0.69	0.068	0.61	0.068	0.56	0.068	0.67	0.068	0.63	0.068	0.464	0.910	0.447	0.383	0.511
*n*-6/*n*-3	9.55	0.52	9.65	0.52	9.55	0.26	9.03	0.34	9.64	0.18	9.56	0.12	0.526	0.454	0.357	0.726	0.738

^1^ *NPD* normal protein diet, *RPD* reduced protein diet, *RPDL* reduced protein diet with leucine addition.

^2^ *TFA* total fatty acids; SFA = 12:0 + 14:0 + 15:0 + 16:0 + 17:0 + 18:0 + 20:0 + 22:0; MUFA = 16:1*c*7 + 16:1*c*9 + 17:1*c*9 + 18:1*c*9 + 18:1*c*11 + 20:1*c*11; PUFA = 18:2*n*-6 + 18:3*n*-3 + 20:2*n*-6 + 20:3*n*-3 + 20:3*n*-6 + 20:4*n*-6 + 20:5*n*-3 + 22:4*n*-6 + 22:5*n*-3 + 22:6*n*-3; *n*-6 PUFA = 18:2*n*-6 + 20:2*n*-6 + 20:3*n*-6 + 20:4*n*-6 + 22:4*n*-6; *n*-3 PUFA = 18:3*n*-3 + 20:3*n*-3 + 20:5*n*-3 + 22:5*n*-3 + 22:6*n*-3.

^3^Total fatty acids are expressed as g/100 g liver; fatty acid composition is expressed as % total fatty acids.

### Gene expression levels of lipogenic enzymes and transcription factors in the liver

**Fig. 1:**
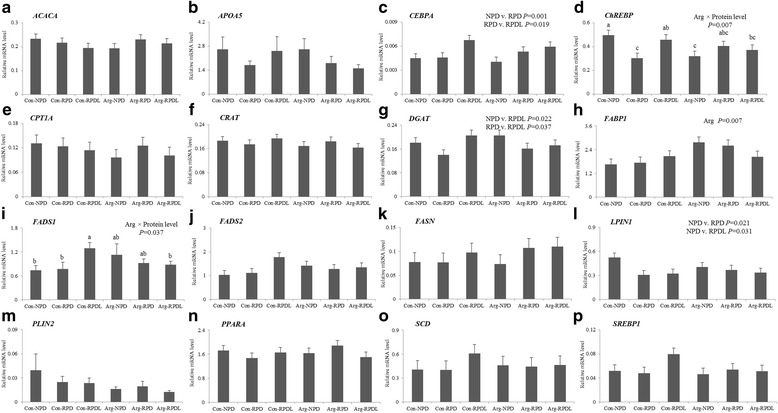
Effect of dietary arginine, leucine and protein level on gene expression in the liver of commercial crossbred pigs: **a** acetyl-CoA carboxylase (*ACACA*), **b** Apolipoprotein A-V (*APOA5*), **c** CCAAT/enhancer binding protein alpha (*CEBPA*), **d** Carbohydrate response element binding protein (*ChREBP*), **e** Carnitine palmitoyltransferase 1A (*CPT1A*), **f** carnitine O-acetyltransferase (*CRAT*), **g** Diacylglycerol acyltransferase (*DGAT*), **h** Fatty acid binding protein 1 (*FABP1*), **i** Fatty acid desaturase 1 (*FADS1*), **j** fatty acid desaturase 2 (*FADS2*), **k** fatty acid synthase (*FASN*), **l** Lipin 1 (*LPIN1*), **m** Perilipin 2 (*PLIN2*), **n** Peroxisome proliferator-activated receptor alpha (*PPARA*), **o** Stearoyl-CoA desaturase (*SCD*), **p** Sterol regulatory element binding protein 1 (*SREBP1*). Con, control diet; NPD, normal protein diet; RPD, reduced protein diet; RPDL, reduced protein diet with leucine addition. Values are means, with standard error represented by vertical bars. ^a, b^ Mean values within a row with unlike letters were significantly different (*P* < 0.05). “Arg” and Arg × protein level mean significant effect of arginine or interaction between arginine and protein level, respectively

The gene expression levels of essential enzymes and transcription factors responsible for lipid metabolism in the liver are presented in Fig. 1. A significant interaction between arginine supplementation and protein level was found for the mRNA levels of *ChREBP* (*P* = 0.007) and *FADS1* (*P* = 0.037). In pigs fed on diets without arginine supplementation, RPD decreased *ChREBP* expression level (*P* = 0.007), and RPDL increased (*P* = 0.037) *FADS1*. RPD increased the expression levels of *CEBPA* (*P* = 0.001), when compared to NPD; also, RPDL increased *CEBPA* when compared to RPD (*P* = 0.019). mRNA levels of *DGAT* were increased in pigs fed on RPDL, relative to NPD (*P* = 0.022) and RPD (*P* = 0.037). The expression levels of *LPIN1* were down-regulated in RPD (*P* = 0.021) and RPDL (*P* = 0.031), when compared to NPD. Arginine, regardless the level of protein in the diets, increased *FABP1* (*P* = 0.007) mRNA levels. *ACACA*, *APOA5*, *CPT1A*, *CRAT*, *FADS2*, *FASN*, *PLIN2*, *PPARA*, *SCD* and *SREBP1* expression levels were kept unchanged by dietary treatments (*P* > 0.05).

### Correlation analysis

Pearson’s correlation coefficients between fatty acids and gene expression levels in the liver are shown in Table 3. The *FADS1* gene was negatively correlated with 18:3*n-3* (*P* < 0.01) and positively associated with 20:1*c*11 (*P* < 0.05). Likewise, *FADS2* relative mRNA levels were negatively correlated with 18:2*n*-6 (*P* < 0.05), and positively correlated with 20:1*c*11 (*P* < 0.05). *FASN* gene was positively correlated with 20:3*n*-6 (*P* < 0.05) and *SCD* with 22:4*n*-6 (*P* < 0.05). *DGAT* expression levels were negatively correlated with 18:2*n*-6 (*P* < 0.01) and 20:2*n*-6 (*P* < 0.05). *PLIN2* mRNA levels correlated positively with 16:1*c*7 (*P* < 0.05) and 18:3*n*-3 (*P* < 0.001). *PPARA* was negatively correlated with 20:3*n*-3 (*P* < 0.05). Finally, *SREBP1* gene was positively correlated with 12:0 (*P* < 0.05) and 20:3*n*-6 (*P* < 0.01), and negatively with 18:1*c*11 (*P* < 0.05).

**Table 3:** Pearson’s correlation coefficients between the fatty acid composition and the relative gene expression levels in the liver from commercial crossbred pigs^1−2^

	*ACACA*	*APOA5*	*CEBPA*	*ChREBP*	*CPT1A*	*CRAT*	*DGAT*	*FABP1*	*FADS1*	*FADS2*	*FASN*	*LPIN1*	*PLIN2*	*PPARA*	*SCD*	*SREBP1*
Fatty acids																
12:0																0.29^*^
14:0																
15:0																
16:0																
16:1*c*7													0.30^*^			
16:1*c*9																
17:0																
17:1*c*9																
18:0																
18:1*c*9																
18:1*c*11																−0.29^*^
18:2*n*-6							−0.35^**^			−0.29^*^						
18:3*n*-3									−0.29^*^				0.51^***^			
20:0																
20:1*c*11									0.27^*^	0.28^*^						
20:2*n*-6							−0.32^*^									
20:3*n*-3														−0.27^*^		
20:3*n*-6											0.31^*^					0.35^**^
20:4*n*-6																
20:5*n*-3																
22:0																
22:4*n*-6															0.28^*^	
22:5*n*-3																
22:6*n*-3																

^1^Statistical significance of Pearson correlation coefficients: ^*^, *P* < 0.05; ^**^, *P* < 0.01; ^***^, *P* < 0.001.

^2^Fatty acid contents expressed as μmol/g liver.

## Discussion

In order to gain insights on the underlying molecular mechanisms that control hepatic lipid metabolism in pigs fed reduced protein diets with amino acids supplementation (arginine and leucine), the gene expression levels of essential lipogenic and lipolytic enzymes and associated transcription factors were evaluated. Furthermore, the effect of dietary arginine and leucine supplementation combined with protein level and molecular mechanisms responsible for fat partitioning between adipose tissue and muscle are available elsewhere [19]. Briefly, diets supplemented with arginine, either alone or in combination with the RPD or leucine, promoted, in contrast to *longissimus lumborum* muscle, a lipogenic effect on adipose tissue. In addition, an increase on IMF content of *longissimus lumborum* muscle was observed in pigs fed on low protein diets [19]. Dietary arginine had no effect on growth performance parameters (ADFI, ADG, and G:F), but when dietary protein level was reduced by 19%, ADG was negatively affected [22]. This is probably explained by the lysine reduction on these diets [23]. Results on pigs’ performance and feed efficiency are published in Madeira et al. [22]. Moreover, entire male pigs were used because these animals are leaner than gilts or castrated barrows, thus having low eating quality, and being the most used in the Portuguese swine industry.

Data presented here indicate that, in the liver, dietary treatments affected more plasma metabolites than fatty acid composition or the transcriptional profile of essential lipogenic and lipolytic enzymes and associated transcription factors. A significant interaction between arginine supplementation and protein level was consistently observed across all plasma lipids. Arginine supplementation in normal protein diet increased plasma lipids, in particular total lipids, total cholesterol, HDL-cholesterol, LDL-cholesterol, VLDL-cholesterol and triacylglycerols. Arginine and reduced protein diets increased individually total lipids but, when combined, a clear decrease in total lipids was observed, suggesting a synergistic effect of both variables. L-arginine has important roles in physiology and overall metabolism; hence, it is beneficial for nutrient metabolism, immune response and circulatory functions in animals and humans [8, 11, 24, 25]. Fatty acid binding protein one (*FABP1*) prevents lipotoxicity of free fatty acids and regulates fatty acid trafficking and partition [26]. Its mRNA expression level was increased with arginine supplementation, which can be related to the increase of total lipids in plasma. Our results indicate that dietary arginine increase concentrations of total lipids, VLDL-cholesterol and triacylglycerols in plasma, which could be associated with increased fat accretion in the carcass [22]. In contrast, Hu et al. [27] reported that arginine supplementation improved nutritional status and lean tissue mass, while beneficially reduced ammonia, free fatty acids, triacylglycerols, and cholesterol levels in the plasma, as well as white fat in the body. In line with Hu et al. [27] and contrasting to our own data, He et al. [28] reported that dietary arginine reduces VLDL-cholesterol, lipids and triacylglycerols concentrations in piglets. Also, Tan et al. [8] reported that 1% of arginine supplemented on diet fed to growing finishing pigs for 60 days reduced positively serum triacylglycerols by 20% and whole-body fat content by 11%, while increasing whole-body skeletal muscle content by 5.5%. The variations of total lipids in plasma and related metabolites support the notion that arginine and its products play an important role in the metabolism of energy substrates [11, 29]. Arginine stimulates the secretion of growth hormone and insulin in mammals, thus playing an important role on the regulation of protein metabolism [9, 30]. In our study, arginine supplementation did not affect plasma hormones, insulin and leptin. Fu et al. [25] reported that arginine increases fatty acid oxidation and glucose in insulin-sensitive tissues, thereby reducing accretion of fat in white adipose tissues. Nevertheless, in our study, arginine supplementation in NPD decreased glucose concentration in plasma. Diacylglycerol acyltransferase (*DGAT*) catalyses the final step in triacylglycerol biosynthesis by converting diacylglycerols and fatty acyl-CoA into triacylglycerols [31]. In our study, leucine increased *DGAT* mRNA expression level but decreased triacylglycerols content which stands out as an apparent contradiction. This remains to be elucidated. Moreover, apoliprotein A-V (*APOA5*) is a key regulator of plasma triacylglycerols and inhibits the production of VLDL-cholesterol, the major carrier of triacylglycerols [32]. Herein, the *APOA5* mRNA expression was unaffected by dietary treatments.

Blat et al. [33] reported that pigs fed on high dietary protein levels relative to normal, had increased insulin levels and consequently, increased HOMA-IR values. Nonetheless, insulin resistance index was found within the normal physiological range accepted for pigs, i.e., below 2.4 [34], even if arginine supplementation decreased HOMA-IR. As being so, this is a finding that does not deserve further pathophysiological understanding. Insulin stimulates fatty acid synthesis which leads to triacylglycerols formation and storage [35]. Accordingly, hepatic total fatty acids tend to decrease with arginine supplementation. Also, arginine affected all plasma hepatic markers, ALT, AST and GGT, but once again, the values observed are within the reference ranges for pig (31–58 U/L for ALT, 32–84 U/L for AST and 10–52 U/L for GGT) [36].

Previous studies have shown that dietary supplementation with arginine reduces plasma concentrations of urea in pigs [37]. Contrarily, in our study, arginine supplementation kept unchanged the urea levels in plasma. It is known that arginine is an intermediate in the urea cycle [27]. Unexpectedly, low protein diets increased urea levels, although the values found are still within the reference range for pig, which is 100–300 mg/L [36], therefore suggesting unaffected renal function.

In our study, leucine supplementation did not affect total cholesterol and LDL-cholesterol, contrarily to results described by Zhang et al. [38]. Those authors [38] reported that leucine supplementation decreases glucose metabolism, reduces diet-induced insulin resistance and lowers plasma glucagon levels and hepatic glucose-6-phosphatase mRNA expression in rats. In our study, leucine supplementation increased plasma glucose in combination with arginine supplementation without affecting insulin.

Dietary treatments had no impact on total fatty acids in the liver, which partially concurs with similar mRNA levels found for stearoyl-CoA desaturase (SCD), one of the key lipogenic enzymes for fatty acid biosynthesis [39]. Together with subcutaneous fat, liver plays an important role in mediating fatty acid metabolism in pigs, mainly triacylglycerols synthesis [40, 41]. As previously reported, dietary arginine did not increase IMF in pigs but enhanced total fat in subcutaneous adipose tissue by 6% [19] in parallel with the up-regulation of the lipogenic enzyme SCD [19]. Our results indicate that mRNA expression level of *FABP1* increased with arginine supplementation, although arginine only increased 15:0 and 20:0 saturated fatty acids in the liver. RPD, with or without leucine supplementation increased CCAAT/enchancer bonding protein alpha (*CEBPA*), that plays a key role in the regulation of adipogenesis and lipogenesis [42] and decreased Lipin 1 (*LPIN1*) mRNA expression levels, that is crucial for adipocyte differentiation, maintenance of mature adipocyte function, and lipogenesis [43, 44]. RPD activated lipogenic mRNA levels and increased IMF content by approximately 45–48% [19]; consequently, RPD up-regulated *CEBPA* in the liver. Likewise, the expression level of *LPIN1* decreased because *LPIN1* is a transcriptional coactivator that promotes fat oxidation and suppresses de novo lipogenesis [45]. However, RPD did not increase total fatty acids in the liver. This finding indicates that low protein diets do not seem to promote fatty liver, a pathophysiological state related to various metabolic disorders, such as obesity, insulin resistance and diabetes, and hyperlipidaemia [41, 46].

The reduction of dietary protein increased oleic acid (18:1*c*9) percentage and tended to increase MUFA proportions (*P* = 0.051) in the liver. This finding was not supported by *SCD* gene expression levels which, as already stated, were unchanged by RPD. SCD is a key enzyme for unsaturated fatty acids biosynthesis by catalysing the 9-*cis* desaturation of saturated fatty acyl-CoA [47]. Conversely, RPD decreased SFA and *n*-3 PUFA percentages in the muscle. Similarly to the liver, RPD enhanced MUFA proportions in subcutaneous adipose tissue, mainly at the expenses of 18:1*c*9 increase [19]. The restriction of dietary protein combined with leucine did not change fatty acid composition in liver and subcutaneous adipose tissue [19].

## Conclusion

A significant interaction between arginine and protein level was determinant on results found for plasma lipids and hepatic markers. Data clearly indicated that the effect of supplemented arginine is dependent on dietary protein level. Specifically, arginine supplemented to a normal protein diet increased total lipids, total cholesterol, HDL-cholesterol, LDL-cholesterol, VLDL-cholesterol and triacylglycerols, but promoted no changes on total fatty acid content in the liver. Hence, arginine does not appear responsible for enhancing hepatic fatty acid deposition. In a similar manner, leucine supplementation and dietary protein reduction promoted no changes on hepatic fatty acid content. Once again, restriction of dietary protein does not seem accountable for hepatic fatty acid deposition. The lack of effect of arginine or dietary protein in the liver is probably directly related to the minor contribution of liver to lipid metabolism in the pig. Ultimately, arginine, leucine and dietary protein reduction do not seem to contribute for fatty liver onset, which is in opposition to the effects previously described in a companion paper for adipose tissue and skeletal muscle [19].

## Methods

### Animals and experimental diets

This experiment was performed at Unidade de Investigação em Produção Animal facilities (Instituto de Investigação Agrária e Veterinária, UIPA-INIAV). All procedures were reviewed by the Ethics Commission of CIISA/FMV and approved by the Animal Care Committee of the National Veterinary Authority (Direcção-Geral de Alimentação e Veterinária, Portugal), in compliance with European Union legislation (2010/63/EU Directive). The staff members responsible for animal experiments hold a certified licence for conducting experiments on live animals from the National Veterinary Authority. Fifty-four commercial crossbred (25% Duroc, 25% Pietrain, 25% Large White and 25% Landrace) entire male pigs were selected with an initial body weight of 58.9 ± 1.59 kg (mean ± standard deviation). Pigs were fed a standard concentrate diet from weaning until the beginning of the experiment. Afterward, pigs were grouped in three pens containing three pigs each with individual control of feed intake (*n* = 9) and randomly assigned to one of the six isoenergetic (14 MJ ME/kg) dietary treatments (isonitrogenous control or arginine treatment, and two protein levels with or without leucine addition). Dietary treatments were, as follows: 16.0% of crude protein (normal protein diet, NPD (based on NRC [48]); 13.0% of crude protein (reduced protein diet, RPD); and 13.0% of crude protein plus L-leucine to achieve 2% (reduced protein diet with leucine, RPDL). Arginine treatment and isonitrogenous control were obtained through supplementation of basal diets with 1.0% of L-arginine and 2.05% of L-alanine, respectively. Arginine or alanine was added to the basal diet at the expense of maize starch to obtain isoenergetic diets. The amino acids were obtained from Fh Diedrichs & Ludwig Post (Mannheim, Germany). During the experiment, pigs were fed individually twice a day and had access to water ad libitum. Feed offered and refusals were recorded daily in order to calculate feed intake. Pigs were weighed weekly, just before feeding, throughout the experiment.

Diets were analysed for dry matter by drying samples at 100 °C to a constant weight. Nitrogen content was determined by the Kjeldahl method [49] and crude protein was calculated as 6.25 × N. Crude fibre was determined by the procedure described by the Association of Official Analytical Chemists (AOAC) [49]. Samples were extracted with petroleum ether, using an automatic Soxhlet extractor (Gerhardt Analytical Systems, Königswinter, Germany) to determine crude fat. Ash and starch contents were quantified, according to the procedures described in AOAC [49] and Clegg [50], respectively. Gross energy in the feed was determined by adiabatic bomb calorimetry (Parr 1261, Parr Instrument Company, Moline, IL, USA). Fatty acid methyl esters (FAME) in feed samples were analysed by one-step extraction and transesterification, using heptadecaenoic acid (17:0) as internal standard [51]. Total amino acids were extracted from the feed according to the method described by AOAC [52] and quantified by HPLC (Agilent 1100, Agilent Technologies, Avondale, PA, USA), including lysine, according to Henderson et al. [53]. The ingredients, chemical composition, amino acid and fatty acid profiles of the experimental diets are presented in Table 4.

**Table 4:** Ingredients and chemical, amino acid and fatty acid compositions of experimental diets^1−4^

	Control			Arginine		
Diets	NPD	RPD	RPDL	NPD	RPD	RPDL
Ingredients (%)						
Maize	62.9	67.3	75.0	63.7	72.3	74.5
Barley	10.0	15.0	8.00	10.0	10.0	10.0
Soybean meal	18.9	10.9	9.60	16.3	7.80	7.2
Sunflower meal	1.64	0.44	-	4.56	4.66	1.98
Soybean oil	1.15	0.98	0.99	1.06	0.88	0.85
Calcium carbonate	0.73	0.73	0.71	0.72	0.70	0.71
Bi-calcium phosphate	1.21	1.32	1.38	1.22	1.35	1.39
Sodium bicarbonate	0.11	0.01	-	0.14	0.06	0.07
Salt	0.35	0.43	0.44	0.33	0.39	0.38
L-Lys	0.30	0.12	0.17	0.34	0.17	0.21
L-Met	0.06	-	-	0.06	-	-
L-Thr	0.07	-	-	0.08	-	-
L-Ala	2.05	2.05	2.05	-	-	-
L-Arg	-	-	-	1.00	1.00	1.00
L-Leu	-	0.17	1.14	-	0.17	1.17
Vitamin-trace mineral premix	0.40	0.40	0.40	0.40	0.40	0.40
Acid mixture	0.10	0.10	0.10	0.10	0.10	0.10
Antioxidant mixture	0.005	0.005	0.005	0.005	0.005	0.005
Chemical composition (% diet)						
DM	87.5	87.7	87.8	87.7	87.7	87.9
Crude protein	16.0	13.1	13.1	15.9	12.9	12.7
Starch	38.3	42.6	42.5	38.5	42.5	43.1
Crude fat	3.36	3.46	3.54	3.46	3.46	3.56
Crude fibre	4.38	3.22	3.06	4.66	4.20	3.36
Ash	3.88	3.78	3.78	4.16	3.98	3.80
Ca	0.66	0.73	0.75	0.59	0.68	0.71
P	0.49	0.51	0.52	0.51	0.52	0.52
ME (MJ ME/kg)	13.8	14.1	14.3	13.9	14.1	14.3
Amino acid composition (% diet)						
Ala	3.13	3.25	3.52	0.16	0.51	0.33
Arg	1.05	0.83	0.49	1.84	1.60	1.56
Asp	0.49	0.35	0.31	0.45	0.38	0.30
Glu	2.07	1.54	1.38	1.82	1.59	1.34
Gly	0.43	0.35	0.41	0.63	0.43	0.41
His	2.02	1.21	0.92	1.27	1.02	0.90
Ile	0.45	0.32	0.38	0.50	0.35	0.35
Leu	1.01	0.93	1.51	0.95	0.94	1.74
Lys	0.84	0.47	0.45	0.70	0.43	0.43
Met	0.02	0.04	0.07	0.06	0.18	0.10
Phe	0.68	0.47	0.28	0.39	0.33	0.31
Pro	0.83	0.79	0.61	0.85	0.96	0.89
Ser	0.81	0.67	0.61	0.78	0.63	0.57
Thr	0.17	0.10	0.12	0.20	0.19	0.18
Tyr	0.31	0.20	0.18	0.24	0.17	0.13
Val	0.70	0.56	0.44	0.57	0.47	0.45
Fatty acid composition (% total fatty acids)						
16:0	15.0	15.3	14.9	15.0	15.0	14.9
18:0	2.72	2.47	2.65	2.58	2.43	2.38
18:1*c*9	24.9	25.0	25.8	24.9	25.4	25.6
18:1*c*11	1.05	0.97	0.98	1.01	0.95	0.94
18:2*n*-6	53.0	53.1	52.8	53.2	53.4	53.3
18:3*n*-3	3.32	3.10	2.85	3.22	2.77	2.77

^1^ *NPD* normal protein diet, *RPD* reduced protein diet, *RPDL* reduced protein diet with leucine addition;

^2^As-fed basis;

^3^ *ME* metabolisable energy;

^4^The list of fatty acids and amino acids presented contains most relevant and usually published.

### Pigs slaughter and sampling

After 17–19 h fasting, pigs were slaughtered at an average body weight of 91.7 ± 1.61 kg at the INIAV experimental abattoir. After electrical stunning and exsanguination, blood was obtained from the jugular vein and collected into tubes containing heparin and centrifuged at 1500 g for 15 min to obtain plasma. Samples for gene expression analysis were collected from the middle lobe of the liver, rinsed with sterile RNAse-free cold saline solution, cut into small pieces (thickness of ~0.3 cm), stabilised in RNA Later® (Qiagen, Hilden, Germany) and kept at −80 °C. For determination of fatty acids, liver samples were vacuum packed and stored at −20 °C, until analysis.

### Plasma metabolites

Total cholesterol, high-density lipoprotein cholesterol (HDL-cholesterol), low-density lipoprotein cholesterol (LDL-cholesterol), triacylglycerols (TAG), phospholipids, total protein, urea, glucose, aspartate aminotransferase (AST), alanine aminotransferase (ALT), gamma-glutamyltransferase (GGT) and alkaline phosphatase (ALP) were analysed through diagnostic kits (Roche Diagnostics, Mannheim, Germany), using a Modular Hitachi Analytical System (Roche Diagnostics). Very low-density lipoprotein cholesterol (VLDL-cholesterol) and total lipids were calculated by Friedewald et al. [54] and Covaci et al. [55] formulas, respectively. Insulin and leptin concentrations were determined through the Porcine Insulin RIA kit (PI-12 K, Linco Research, Millipore, MA, USA) and the Multi-Species Leptin RIA kit (XL-85 K, Linco Research), respectively. To calculate the degree of insulin resistance, it was used the homeostasis model assessment using the insulin resistance index (HOMA-IR): fasting plasma glucose (mmol/L) times fasting plasma insulin (mU/L) divided by 22.5 [56]. Low HOMA-IR values indicate high insulin sensitivity, while high HOMA-IR values indicate high insulin resistance [56].

### Hepatic lipid extraction and fatty acid composition

Liver samples were lyophilised (−60 °C and 2.0 hPa), maintained exsiccated at RT, and analysed within 2 weeks. Total lipids were extracted in duplicate and gravimetrically measured, according to Folch et al. [57], using dichloromethane and methanol (2:1 *v*/v) as substitute of chloroform and methanol (2:1 *v*/v), as described by Carlson [58]. Fatty acids were converted to methyl esters (FAME) by a combined transesterification procedure using NaOH in anhydrous methanol (0.5 M), followed by HCl:methanol (1:1 *v*/v), at 50 °C during 30 and 10 min, respectively, according to Raes et al. [59] protocol. FAME were determined by gas chromatograph (HP6890A, Hewlett–Packard, PA, USA), equipped with a flame ionization detector (FID) and a capillary column (CP-Sil 88; 100 m × 0.25 mm i.d., 0.20 μm film thickness; Chrompack, Varian Inc., Walnut Creek, CA, USA), asin Alves and Bessa [60]. The quantification of total FAME was carried out using nonadecanoic acid methyl ester (19:0) as the internal standard and by the conversion of relative peak areas into weight percentages. Fatty acids were identified by their retention times, corresponding to their standards from Supelco Inc. (Bellefonte, PA, USA). Fatty acids were expressed as g/100 g of total fatty acids.

### RNA isolation and cDNA synthesis

A modified protocol combining Trizol (Invitrogen, CA, UK) and RNeasy Mini kit (Qiagen, Hilden, Germany) was used to isolate and purify total RNA from the liver. RNA samples were treated with DNAse I (Qiagen), prior to RT-qPCR. All procedures were performed according to manufacturer’s instructions. RNA was quantified using a NanoDrop ND-2000c spectrophotometer (Nanodrop, Thermo Fisher Scientific, Willmington, DE, USA). A260/280 ratios were between 1.9 and 2.1. A High-Capacity cDNA Reverse Transcription Kit (Applied Biosystems, Foster City, CA, USA) performed the reverse transcription. In brief, each 20 μL RT reaction containing 1 μg of DNase-treated total RNA template, 50 nM random RT Primer, 1× RT buffer, 0.25 mM of each dNTP, 3.33 U/μL multiscribe reverse transcriptase and 0.25 U/μL RNase inhibitor, and it was submitted to 25 °C for 10 min, 37 °C for 120 min and 85 °C for 5 min. The cDNA obtained was divided into aliquots and stored at −20 °C, until analysis.

### Real-time quantitative PCR (RT-qPCR)

Gene specific intron-spanning primers were designed using Primer3 (http://bioinfo.ut.ee/primer3-0.4.0/) and Primer Express Software v. 2.0 (Applied Biosystems, Foster City, CA, USA) based on *Sus scrofa* species sequences (www.ncbi.nlm.nih.gov). Primers were acquired from NZYTech (Lisbon, Portugal). Sequence homology searches against the database of GenBank confirmed that these primers matched only the sequence for which they were designed. The amplicon length ranged between 67 and 166 bp, to ensure optimal DNA polymerization efficiency. In order to test the primers and verify the amplified products, a conventional PCR was performed for all genes, before qPCR experiments. PCR products were sequenced and homology searches were carried out with Blast (www.ncbi.nlm.nih.gov/blast), in order to confirm the identity of amplified fragments. Aiming to find the most stable endogenous control for the liver, five frequently used housekeeping genes, glyceraldehyde-3-phosphate dehydrogenase (*GAPDH*), 60S ribosomal protein L27 (*RPL27*), ornithine decarboxylase antizyme 1 (*OAZ1*), ribosomal protein large P0 (*RPLP0*) and 40S ribosomal protein S29 (*RPS29*) were applied to normalise the results of target genes. The geNorm [61] and NormFinder [62] software packages were used to analyse the expression level stability of housekeeping genes, as described in their manuals. The *RPLP0* and *RPL27* genes were chosen as the most stable internal controls pair for normalization. The sequence of primers, GenBank accession numbers, and product sizes are detailed in Table 5. The StepOnePlus PCR System software (Applied Biosystems) was used to calculate the PCR efficiency for each amplicon, by amplifying 5-fold serial dilutions of pooled cDNA and run in triplicate. All primer sets showed an efficiency between 90 and 110% and correlation coefficients were higher than 0.99. qPCR reactions were performed using MicroAmp Optical 96-well plates (Applied Biosystems) in a StepOnePlus thermocycler (Applied Biosystems) with standard cycling conditions. The 12.5 μl PCR reaction mixture included 6.25 μl of 2× Power SYBR Green PCR Master Mix (Applied Biosystems), 160 nM of forward and reverse primers, and 2 μl of diluted cDNA as template. No transcription and template samples were used as controls. The primer specificity and formation of primer-dimers were checked by melting curve analysis and agarose gel electrophoresis. All analyses were carried out in duplicate, and relative amounts for each target gene were calculated using the geometric mean of *RPLP0/RPL27* as normaliser. The relative expression levels were calculated as a variation of the Livak method [63], corrected for variation in amplification efficiency, as described by Fleige et al. [64].

**Table 5:** Characterization of the select genes used in real time quantitative PCR^1−2^

Gene symbol	Full gene name	GenBank accession number	Forward primer	Reverse primer	Product size (bp)
*ACACA*	Acetyl-CoA carboxylase alpha	NM_001114269.1	ggccatcaaggacttcaacc	acgatgtaagcgccgaactt	120
*APOA5*	Apolipoprotein A-V	NM_001159308.1	agggaaaggcttctgggacta	tgtctttcagtctcgtgggctc	107
*CEBPA*	CCAAT/enhancer binding protein (C/EBP) alpha	XM_003127015.2	ggccagcacacacacattaga	cccccaaagaagagaaccaag	71
*ChREBP*	Carbohydrate response element binding protein	XM_003481002.2	tgacatgatccagcctgacc	gggggctcagagaagtttga	126
*CPT1A*	Carnitine palmitoyltransferase 1A	NM_001129805.1	cgattatccaccagccagac	caccccataaccatcgtcag	120
*CRAT*	Carnitine O-acetyltransferase	NM_001113047.1	ggcccaccgagcctacac	atggcgatggcgtaggag	138
*DGAT*	Diacylglycerol acyltransferase	NM_214051.1	caactaccgtggcatcctga	tagaaacagccgtgcattgc	67
*FABP1*	Fatty acid binding protein 1	NM_001004046.1	aacttctccggcaaataccaa	attctgcacgatttccgatg	129
*FADS1*	Fatty acid desaturase 1	NM_001113041.1	gtgggtggacttggcctg	gatgtgcatggggatgtggt	166
*FADS2*	Fatty acid desaturase 2	NM_001171750.1	gccttacaaccaccagcatga	aggccaagtccacccagtc	122
*FASN*	Fatty acid synthase	NM_001099930.1	acaccttcgtgctggcctac	atgtcggtgaactgctgcac	112
*LPIN1*	Lipin 1	NM_001130734.1	aagtcgccgccctgtatttc	ttgtcgctggcctgttttgt	67
*PLIN2*	Perilipin 2	NM_214200.2	catgtccggtgctctcccta	cccagtcacagcccctttag	160
*PPARA*	Peroxisome proliferator-activated receptor alpha	NM_001044526.1	tttccctctttgtggctgct	ggggtggttggtctgcaag	128
*SCD*	Stearoyl-CoA desaturase	NM_213781.1	agccgagaagctggtgatgt	gaagaaaggtggcgacgaac	140
*SREBP1*	Sterol regulatory element binding protein 1	NM_214157.1	gtgctggcggaggtctatgt	aggaagaagcgggtcagaaag	86
*RPLP0* ^2^	Ribosomal phosphoprotein large P0 subunit	NM_001098598.1	tccaggctttaggcatcacc	ggctcccactttgtctccag	95
*RPL27* ^2^	Ribosomal protein L27	NM_001097479.1	gtactccgtggatatccccttg	aacttgaccttggcctctcga	102

^1^Entrez Gene, National Center for Biotechnology Information (*NCBI*)

^2^housekeeping gene

### Statistics

Data were checked for normal distribution and variance homogeneity. As variance heterogeneity was found for the majority of plasma metabolites, fatty acids and genes, these data were analysed using the MIXED procedure in Statistical Analysis Systems software package, version 9.2 (SAS Institute, Cary, NC, USA). The experimental unit was the animal. The model included as fixed effects dietary arginine and the basal diet (protein level with or without leucine supplementation) and their respective interaction, and the REPEATED statement considering the group option to accommodate variance heterogeneity. If the interaction between dietary arginine and protein level was significant, multiple comparisons of least-square means were determined using the PDIFF with Tukey-Kramer adjustment options of SAS. The contrasts between dietary protein level and leucine (NPD vs. RPD, NPD vs. RPDL, and RPD vs. RPDL) were performed. Pearson correlation matrices were computed using the PROC CORR of SAS. The level of significance was set at *P* < 0.05.

## Abbreviations

*ACACA*: Acetyl-CoA carboxylase
*APOA5*: Apolipoprotein A-V
*CEBPA*: CCAAT/enhancer binding protein alpha
*ChREBP*: Carbohydrate response element binding protein
*CPT1A*: Carnitine palmitoyltransferase 1A
*CRAT*: Carnitine O-acetyltransferase
*DGAT*: Diacylglycerol O-acyltransferase
*FABP1*: Fatty acid binding protein 1
*FADS1*: Fatty acid desaturase 1
*FADS2*: Fatty acid desaturase 2
*FAME*: Fatty acid methyl esters
*FASN*: Fatty acid synthase
*GAPDH*: Glyceraldehyde-3-phosphate dehydrogenase
*LPIN1*: Lipin 1
*MUFA*: Monounsaturated fatty acids
*OAZ1*: Ornithine decarboxylase antizyme 1
*PLIN2*: Perilipin 2
*PPARA*: Peroxisome proliferator-activated receptor alpha
*PUFA*: Polyunsaturated fatty acids
RPD: Reduced protein diet
RPDL: Reduced protein diet with leucine supplementation
*RPLP0*: Ribosomal protein large P0
*RPS29*: 40S ribosomal protein S29
*SCD*: Stearoyl-CoA desaturase
*SFA*: Saturated fatty acids
*SREBP1*: Sterol regulatory element binding protein 1
